# Remote Photocatalytic Eradication of Biorecalcitrant Microorganisms via BiOCl_0.2_Br_0.8_—The Applied Aspects of Visible Light-Driven Photocatalysis

**DOI:** 10.1021/acsomega.2c01502

**Published:** 2022-08-15

**Authors:** Razan Abbasi, Hani Gnayem, Yoel Sasson

**Affiliations:** Casali Center of Applied Chemistry, Institute of Chemistry, The Hebrew University of Jerusalem, Jerusalem 9190401, Israel

## Abstract

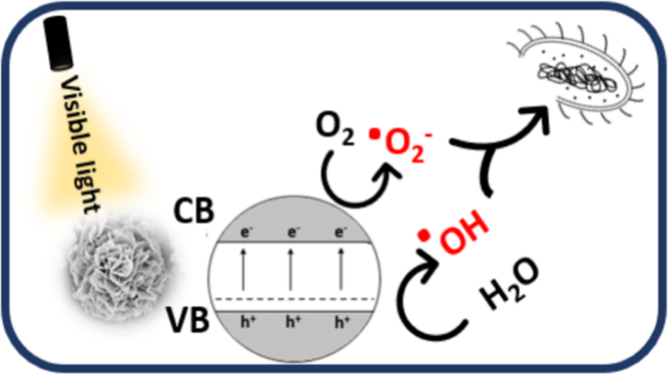

Photocatalysis has an exceptional capacity to eliminate a wide range of harmful microorganisms and is proven to be superior over commonly used disinfection methods. A visible light-induced photocatalyst, the BiOCl_0.2_Br_0.8_@gypsum hybrid composite, composed of microspheres (∼3 μm) molded with a gypsum composite as a honeycomb-shaped filter was proven to inactivate a large selection of bacteria including *Salmonella typhi*, *Bacillus subtilis,* and *Listeria monocytogenes* via remote photocatalysis. The chemical composition and morphology of the composite were unveiled with the help of scanning electron microscopy, transmission electron microscopy, N_2_ sorption, Fourier transform infrared spectroscopy, diffuse reflectance spectroscopy, X-ray diffraction, and X-ray photoelectron spectroscopy. After 6 h under ambient conditions, our system declined the number of viable bacteria by fourfold. A similar effect was observed at a low temperature, where we rapidly and completely diminished *L. monocytogenes* inside a refrigerator within 24 h of visible light illumination.

## 1 Introduction

Environmental contamination is a major challenge causing numerous problems including infectious diseases.^1^ The large number of disease outbreaks due to microorganisms such as bacteria and viruses has drawn attention to the significance of uncovering effective solutions.

Unfortunately, commonly used disinfecting methods such as chlorination and UV radiation have many downsides, including being carcinogenic and environmentally harmful.

Treating harmful bacteria via traditional antibiotics is not much effective, thus raising the demand for a better and safer control of the infectious pathogens.^2^

Photocatalytic oxidation processes are considered among the most promising solutions where titanium dioxide (TiO_2_) has been the utmost studied photocatalyst for over 20 years.^3^

TiO_2_ is nontoxic and has been used as an additive in various applications, including in cosmetics and pharmaceuticals. It also functions as a photocatalyst, widely utilized in self-cleaning coatings,^4,5^ although its wide band gap (3.2 eV) limits its activity to the UV region, restraining its application.^6^ Substances with the ability to function with indoor light sources are of vital importance, especially for pathogen elimination.^7^

Doping of TiO_2_ with other elements^8−10^ or exposing its active facets^11^ can extend the light absorption of TiO_2_ beyond the UV region. However, fabrication of efficient visible light-driven photocatalysts has not yet been fully realized. Bismuth oxyhalides (BiOX; X = Cl, Br, I), which have outstanding visible light photocatalytic activity,^12−16^ have gained substantial attention recently. They consist of a layered crystal structure composed of tetragonal [Bi_2_O_2_]^2+^ positive slabs interweaved by double negative slabs of halogen atoms along the *c* axis.^17^ These unique semiconductors are prevalent in various applications including pigments^18^ and catalysts^19^ for their exceptional optical, light harvesting, and electrical properties.^20−22^

Composite materials BiOX/BiOY, with X = Y = F, Cl, Br, or I, exhibit enhanced visible light photoactivity. Since oxyhalides such as BiOI and BiOBr can absorb visible light and act as a sensitizer, they are able to easily transfer photoinduced electrons on their surface to the conduction band (CB) of BiOCl, for instance, thus leaving holes in the BiOI (or BiOBr) valence band (VB). In this mechanism, the photoinduced electron–hole pairs can be effectively detached, preventing the undesired recombination and thus maximizing the photocatalytic activity.^23^

Several authors reported the unique photochemical activity of mixed oxyhalide derivatives of bismuth. Wang et al.^24,25^ reported the synthesis of BiOI_1–*x*_Cl _*x*_ and BiOI_1–*x*_Br_*x*_ phases which demonstrated high photocatalytic activity under visible light irradiation for the degradation of methyl orange. Chen et al.^23^ reported the highest decomposition rate of rhodamine B with a 70% BiOCl/BiOI composite while that of methyl orange was observed with 20% BiOCl/BiOI, suggesting different degradation mechanisms.

The preparation, characterization, and photoactivity of the novel family of bismuth mixed oxyhalides with the general structure BiOCl_1–*x*_Br_*x*_ were first reported by our lab.^26,27^

The visible light (λ > 420 nm) photocatalytic activity of this family was exhibited via the decomposition of rhodamine B and of acetophenone, as well as by the photo-oxidation of potassium iodide to iodine.

Liu et al. verified again the critical role of the Cl/Br ratio in the unique composite material BiOCl/BiOBr in controlling its photocatalytic activity, that is, the BiOCl_1–*x*_Br_*x*_ heterojunction system demonstrated a higher photocatalytic capacity compared to its individual BiOCl/Br components.^28^

Bhachu et al. reported the fabrication of bismuth oxyhalides as thin films using an aerosol-assisted chemical vapor deposition (CVD) method.^29^ Thin films were also synthesized by a sol–gel technique as reported by Shen et al.^30^ Diverse matrices and techniques were studied aiming to find an ideal way for incorporating bismuth oxyhalides in disinfection and purification applications.

The embedding of bismuth oxyhalides into an inorganic matrix such as gypsum, a natural, environmentally friendly, and fireproof material, has major benefits including the fact that the catalyst–gypsum interactions are based on physical forces, eliminating the need of a binding agent. This formulation also reduces the loss of free surface area and maintains high photocatalytic activity.

In this work, we report a straightforward method for fabricating a bismuth oxyhalides@gypsum-based filter made with green materials.

The filter showed remarkable photocatalytic efficacy and stability in removal and absolute elimination of bacteria such as *Bacillus subtilis*, *Salmonella,* and *Listeria monocytogenes*.

This work emphasizes the applied aspect of photocatalysis, interpreted by the novel concept of remote disinfection of surfaces contaminated by various representative bacteria.

## 2 Methods

### 2.1 Materials

All materials were purchased from Sigma-Aldrich with a purity of ≥98% and were used without further purification.

### 2.2 Bacterial Strains and the Culture Method

The starters of *Salmonella typhimurium* ATCC 14028, *B. subtilis* ATCC 6633, and *L. monocytogenes* ATCC 19114 were grown in tryptic soy broth (TSB) overnight at 35 °C. Tests were performed under sterile conditions, in the case of *S. typhimurium* and *B. subtilis* at room temperature, and at 4 °C in the case of *L. monocytogenes*. 0.2 mL of each starter was inserted to microscope glass slides.

At predetermined intervals, after the beginning of each test, the glass slides were taken out of the chamber or the refrigerator (in the case of *Listeria*), and the bacterial count was performed by inserting each glass slide into a 50 mL tube and washing it with 2 mL of phosphate-buffered saline (PBS) buffer, after which vortexing (at 12,000 rpm, for 1.5 min) and bacterial dilutions were performed; finally, growth via incubation for 24 h in standard methods agar (SMA) plates at 35 °C using the pour plate method enabled the counting of the viable cells.

### 2.3 Synthesis of BiOCl_0.2_Br_0.8_

9.2 g of 19 mmol bismuth nitrate was dissolved in 40 mL of deionized water and 40 mL of glacial acetic acid in a 250 mL beaker and stirred at room temperature until the formation of a transparent, clear solution, typically after 15 min of stirring.

In one batch, 5.518 g of 15.2 mmol cetyltrimethylammonium bromide (CTAB) dissolved in 25 mL of ethanol and 10 mL of deionized water and 4.845 g of 3.8 mmol 25 wt % aqueous solution of cetyltrimethylammonium chloride (CTAC) were added to the above solution with an additional 60 min stirring at room temperature. Filtration of the precipitate was performed, followed by five washes with 50 mL of ethanol and another five washes with 200 mL of deionized water for the removal of nonreactive organic species. The yellowish-white solid (Figure S1) was then dried (in air) before use.

### 2.4 Gypsum Composites

An aqueous dispersion of the photocatalyst (1 g of the photocatalyst in a solution of 7 mL of double-distilled water and 3 mL of ethanol) was employed to spray-coat an elastic mold consisting of hexagons, where the mold’s dimensions were 10 cm × 10 cm × 3 cm with 5 mm hexagonal openings. This process was repeated twice in order to maximize the final loading of the photocatalyst.

Commercially available gypsum was mixed with a stoichiometric amount of distilled water. The curing gypsum–water mixture was poured into the hexagonal-shaped mold, resulting in a honeycomb-shaped filter, including a final photocatalyst load of about 1.8 g, as shown in Figure S2 in the Supporting Information. The BiOCl_0.2_Br_0.8_ density in the composite is 0.006 g/cm^3^.

The filter was reused for several cycles without any significant deterioration of the original activity. It is of utmost importance to mention that each performance test has been repeated three times.

### 2.5 Photocatalytic Reactor

**Figure 1:**
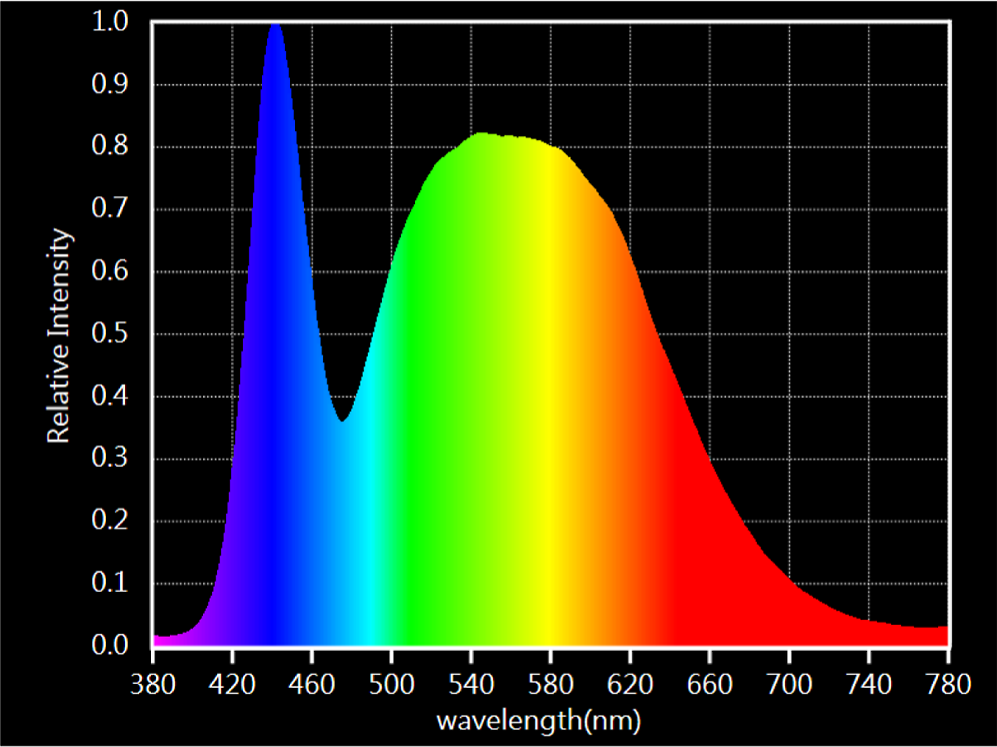
Visible light spectrum emitted by the 10 W white LED (6000 K) lamp.

The device (Figure S3) contained the honeycomb-shaped filter of the gypsum–photocatalyst composite, an LED 10 W lamp as a light source (the light spectrum is shown in Figure 1), and a fan (NMB, model 3610KL-04W-B50), producing the airflow and thus facilitating the photocatalytic activity of BiOCl_0.2_Br_0.8_.

### 2.6 Characterization

A diffractometer (D8 advance, Bruker AXS, Karlsruhe, Germany) was utilized to obtain the X-ray powder diffraction measurements, with a goniometer radius 217.5 mm, a secondary graphite monochromator, 2° Sollers slits, and a 0.2 mm receiving slit. A 5 to 70° 2θ range of X-ray diffraction (XRD) patterns were recorded by employing Cu Kα radiation (λ = 1.5418 Å) under the following measurement conditions: a tube voltage of 40 kV, a tube current of 40 mA, the step-scan mode with a step size of 0.02° 2θ, and a counting time of 1 s per step, at room temperature. The BiOCl_0.2_Br_0.8_-coated gypsum sample was placed on a sample stage that is regulated along the vertical axis.

With the assistance of an environmental scanning electron microscope (Quanta 200, FEI, Eindhoven, the Netherlands), supplied with an energy-dispersive X-ray detector (EDAX-TSL, USA) and activated in the low vacuum mode (0.6 Torr pressure) at a 20 kV accelerating voltage, as well as a high-resolution scanning electron microscope (Sirion, FEI, Eindhoven, the Netherlands) in the ultra-high-resolution mode with a through the lens detector, chemical and morphological analyses were performed.

X-ray photoelectron spectroscopy (XPS) analysis was conducted using an XPS Kratos Axis Ultra (Kratos Analytical Ltd., UK) high-resolution photoelectron spectroscopy instrument.

Transmission electron microscopy (TEM) imaging, high-resolution scanning TEM (STEM) imaging, and elemental mapping were carried out using a Themis Z aberration-corrected scanning transmission electron microscope (Thermo Fisher Scientific) operated at 300 kV and equipped with a Ceta camera, HAADF detector for STEM, and Super-X energy-dispersive X-ray detector for elemental analysis.

The surface area and pore radius were determined by the N_2_ Brunauer–Emmett–Teller (BET) method (NOVA-1200e) and the Barrett–Joyner–Halenda (BJH) method, respectively.

Diffuse reflectance analysis was carried out using a UV–vis spectrophotometer equipped with an Integrating Sphere (JASCO V-650 Series ISV-722).

Fourier transform infrared (FTIR) spectra were collected using a Bruker (Alpha-T) spectrometer.

The visible light spectrum emitted from the 10 W white LED was measured using a spectrophotometer supplied by the “Asensetek” company, model no. ALP-01.

## 3 Results and Discussion

### 3.1 Characterization

**Figure 2:**
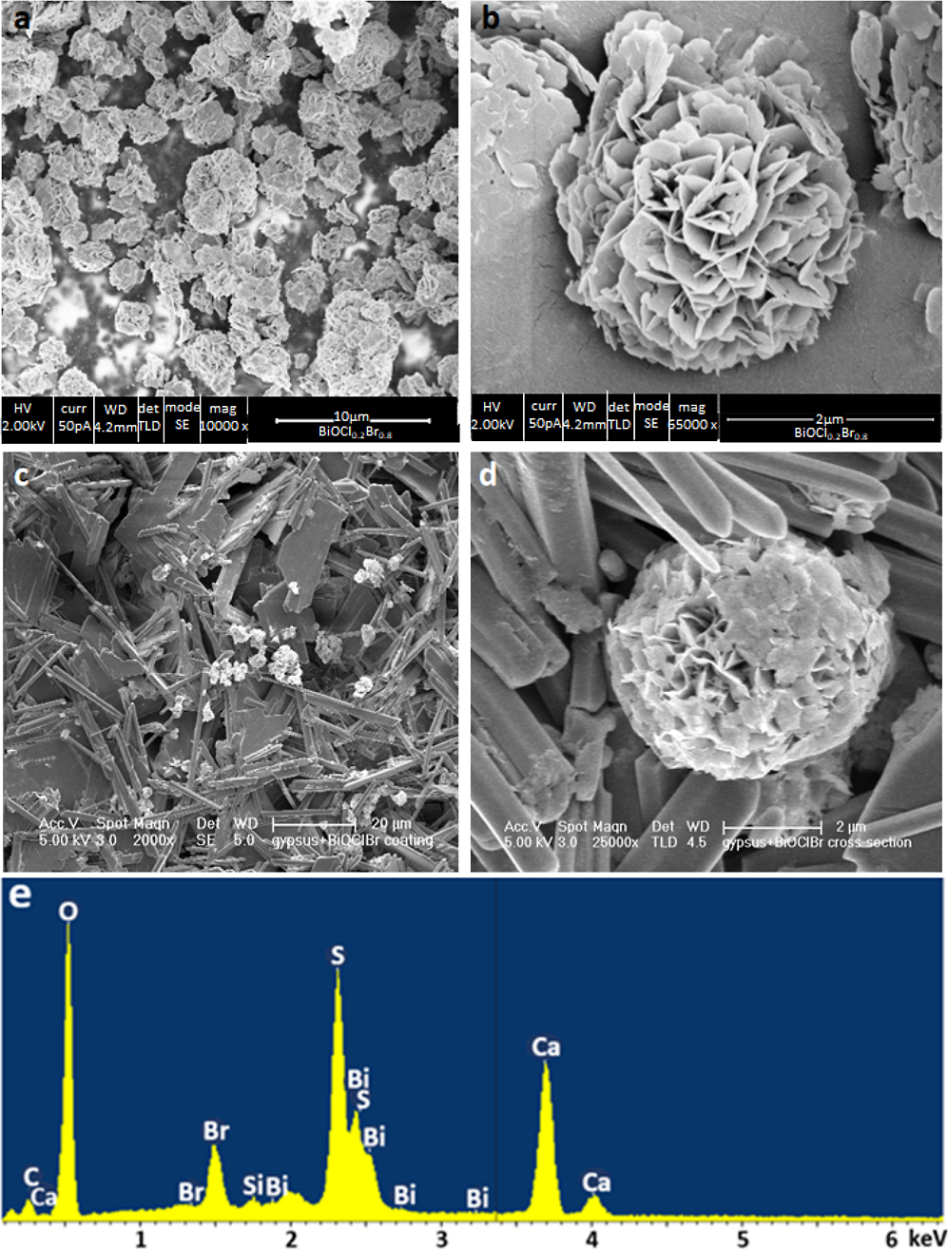
SEM images acquired from dry powders of the as-synthesized BiOCl_0.2_Br_0.8_ (a,b) and from the coated gypsum surface (c,d). The EDS spectrum (e) was acquired from the coated surface at a 30 kV accelerating voltage.

Scanning electron microscopy (SEM) images of the dry as-synthesized BiOCl_0.2_Br_0.8_ as well as of the same material coated on the gypsum surface are shown in Figure 2.

Samples’ morphologies as well as their topographical details were analyzed using high-resolution SEM (HR SEM). BiOCl_0.2_Br_0.8_ particles are about 3 μm in diameter; they are made of thin plates having lateral dimensions of hundreds of nanometers, forming microspheres. The plate’s thickness is about 10 nm. Figure 2c,d shows the BiOCl_0.2_Br_0.8_ microspheres on the gypsum surface. The material’s chemical composition was confirmed from the presented EDS spectrum (Figure 2e), where the elements’ atomic percentages are shown in Table S1, and the elemental mapping of the different elements of the BiOCl_0.2_Br_0.8_ microsphere is shown in Figure S4.

**Figure 3:**
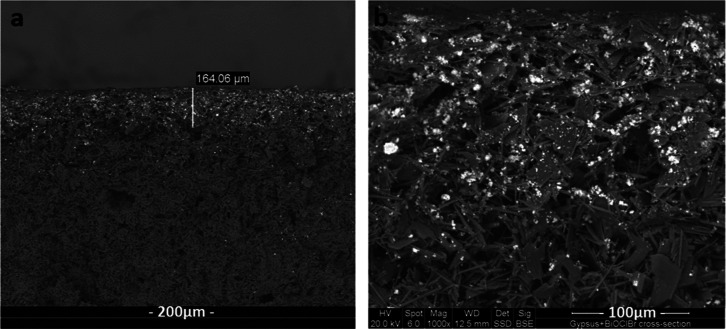
SEM images attained from the cross-sectional sample (a) as well as the top view (b) of the BiOCl_0.2_Br_0.8_-coated gypsum surface.

The cross-section of the sample of the coated gypsum obtained via SEM shown in Figure 3 reveals the position of BiOCl_0.2_Br_0.8_. The photocatalyst is found in the superficial layer of the gypsum, while it is absent in the bulk, as can be seen by comparing with the SEM images of the pure gypsum with no photocatalyst (Figure S5). The coating layer thickness is around 160 μm as shown in Figure 3a, and the microspheres of BiOCl_0.2_Br_0.8_ are micron-sized, displaying a clear contrast in backscattered electron imaging.

**Figure 4:**
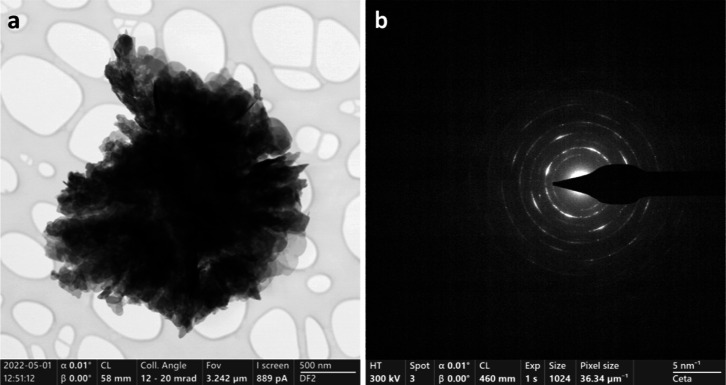
TEM image acquired from the dry powder of the as-synthesized BiOCl_0.2_Br_0.8_ (a) and the corresponding electron diffraction pattern (b).

To confirm the morphology and to further investigate the detailed microstructure of BiOCl_0.2_Br_0.8_, TEM imaging was carried out. A microsphere formed from the assembly of thin plates is shown in Figure 4a, consistent with the SEM observation. The selected area electron diffraction pattern (Figure 4b) of the BiOCl_0.2_Br_0.8_ microsphere in Figure 4a confirms that the material is polycrystalline in nature.

**Figure 5:**
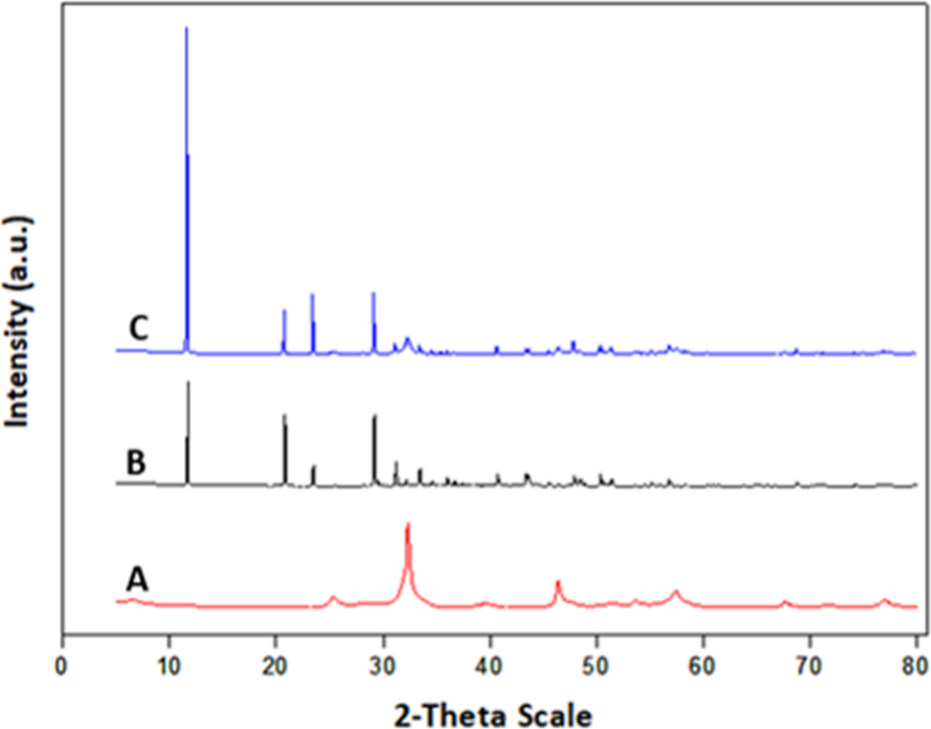
XRD patterns acquired from the as-synthesized BiOCl_0.2_Br_0.8_ nanoparticles (A), pure gypsum (B), and BiOCl_0.2_Br_0.8_ layer on gypsum (C).

The composition of BiOCl_0.2_Br_0.8_ was determined by XRD measurements, where the patterns attained from the as-synthesized BiOCl_0.2_Br_0.8_, from the BiOCl_0.2_Br_0.8_ layer on gypsum, and from pure gypsum for the purpose of comparison are shown in Figure 5.

The diffraction peaks of both BiOCl_0.2_Br_0.8_ and gypsum phases are clearly observable in Figure 5c. One of the crystal phases is BiOCl_0.2_Br_0.8_ [unit cell parameters *a* = 3.91 Å, *c* = 7.94 Å; *P*4/*nmm* (no. 129) space group], while the second phase is gypsum Ca(SO_4_)·2(H_2_O).

As elucidated in our previous studies,^26^ using the value of the c parameter acquired from XRD data via *x* = 10.966–1.354*c*, where *x* is the chlorine content, the photocatalyst composition was determined. *x* was found to be 0.21, similar to the claimed photocatalyst composition.

The photocatalyst crystallites are nanosized, as shown by their broad peaks in Figure 5a. Using the Scherrer equation, the crystallite sizes of the photocatalyst were calculated to be about 12 nm.

**Figure 6:**
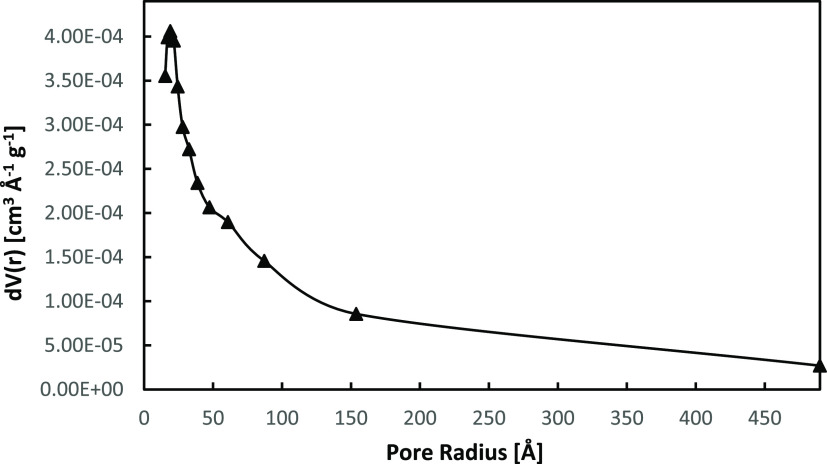
BJH pore size distribution of the pure BiOCl_0.2_Br_0.8_ powder.

The surface chemical composition of the as-synthesized BiOCl_0.2_Br_0.8_ was studied using XPS. The photocatalyst sample was found to be composed of Bi, O, Cl, and Br (Table 1), having a surface area of 11.856 m^2^/g and a pore radius of 18.96 Å (Figure 6). The resultant BET isotherm of the as-synthesized BiOCl_0.2_Br_0.8_ is shown in the Supporting Information (Figure S6).

**Table 1:** Atomic and Mass Concentration Table as Measured by XPS

	atomic conc. [%]	error [%]	mass conc. [%]	error [%]
Bi 4f	21.78	0.97	67.94	0.67
Cl 2p	2.63	0.58	1.39	0.31
O 1s	24.59	1.81	5.87	0.46
Br 3d	15.35	0.7	18.32	0.25
C 1s	33.02	2.51	5.92	0.59
N 1s	2.62	1.76	0.55	0.37

**Figure 7:**
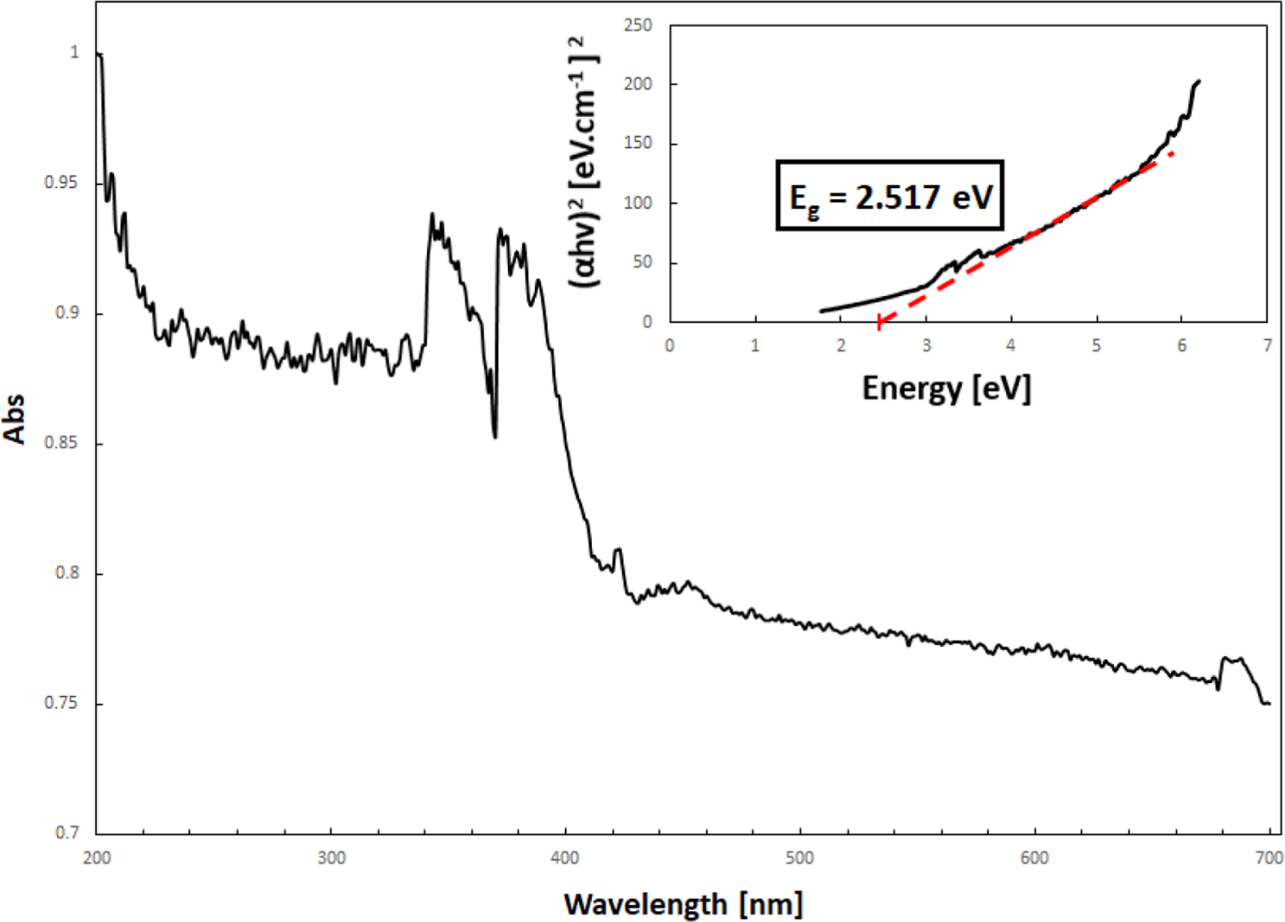
DRS spectrum and Tauc’s plot of the as-synthesized BiOCl_0.2_Br_0.8_.

The diffuse reflectance spectrum was collected to calculate the band gap of BiOCl_0.2_Br_0.8_ via Tauc’s plot (Figure 7), an important physical variable to the photocatalytic activity of semiconductors. The calculated value was 2.517 eV, from which we could determine both the CB and VB positions, as both are critical for the understanding of the photodegradation mechanism of pollutants. The VB and CB edge positions of BiOCl_0.2_Br_0.8_ were calculated using the following equations^23^
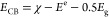

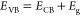
where χ is the electronegativity of the semiconductor, *E*^e^ is the energy of free electrons on the hydrogen scale (≈4.5 eV), and *E*_g_ is the band gap energy of the semiconductor. The values of electronegativity (χ), band gap energy (*E*_g_), and the corresponding CB (*E*_CB_) and VB (*E*_VB_) calculated from the diffuse reflectance spectrum for the as-synthesized BiOCl_0.2_Br_0.8_ photocatalyst are 6.21, 2.52, 0.45, and 2.97 eV, respectively.

**Figure 8:**
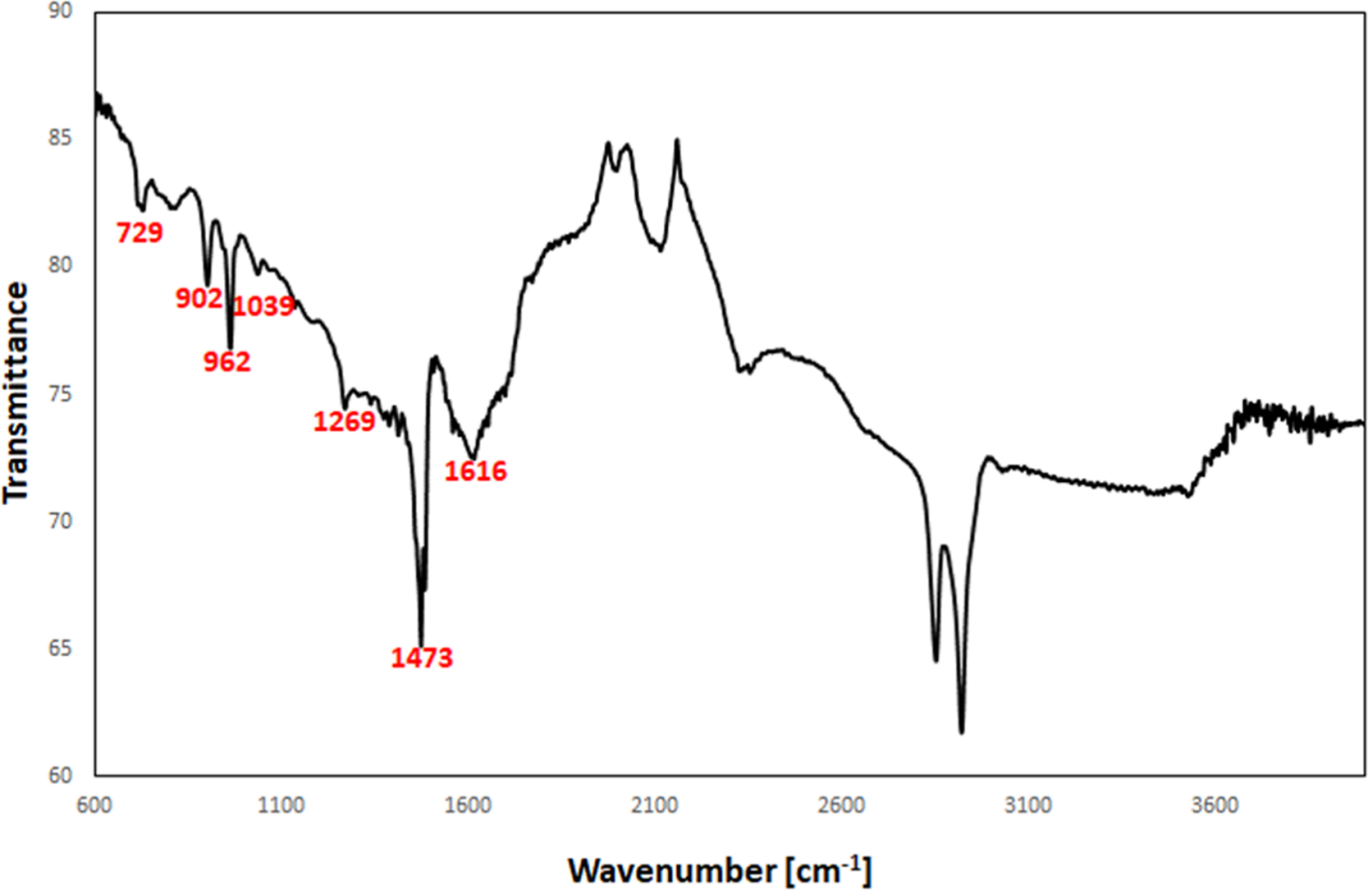
FTIR spectrum of the as-synthesized BiOCl_0.2_Br_0.8_.

The functional groups in the as-synthesized BiOCl_0.2_Br_0.8_ were studied using FTIR spectroscopy (Figure 8). The peaks below 1000 cm^–1^ are attributed to Bi–O bonds^31^ whereas those in the range of 1000–1500 cm^–1^ may belong to both the Bi–Br band^32^ and Bi–Cl. The asymmetric and symmetric stretching vibration peaks of Bi–Cl can be observed at 1039 and 1473 cm^–1^, respectively.^32,33^

### 3.2 Bactericidal Activity

The bactericidal activity of BiOCl_0.2_Br_0.8_ was investigated using the model organisms *S. typhimurium*, *B. subtilis,* and *L. monocytogenes* to represent a variety of common bacteria.

For *S. typhimurium* and *B. subtilis,* tests were performed at room temperature under sterile conditions by contaminating chosen surfaces (microscope glass slides) using a bacterial suspension in a closed 70 L plastic chamber.

The chamber contained both the photocatalytic reactor and the contaminated glass slides which were laid on an empty Petri dish at a 15 cm distance from the reactor.

The atmosphere at all points inside the chamber was equivalent since the bacterial count results were not affected when the contaminated glass slide was placed in front of the photocatalytic reactor versus on its side.

The photocatalytic activity of the hybrid composite commenced after turning on both the LED light and the fan of the photocatalytic system.

At predetermined intervals, after the beginning of each test, the glass slides were taken out of the chamber, and the bacterial counting was performed after bacterial dilutions and growth via incubation using agar plates.

**Figure 9:**
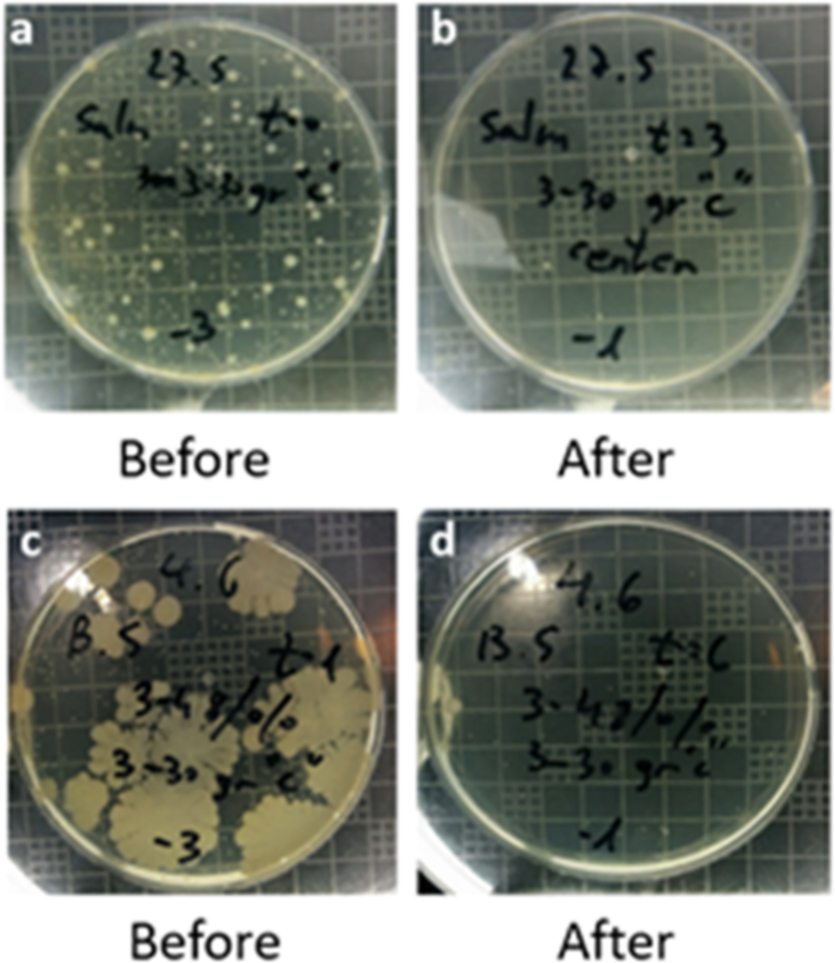
Decrease in the number of viable *S. typhimurium* (a,b) and *B. subtilis* (c,d) bacteria after exposure to the treated and oxidant-enriched air as a function of time.

Table 2 shows the bacterial viability as a function of time after visible light illumination of BiOCl_0.2_Br_0.8_. The bacterial growth of both *B. subtilis* and *S. typhimurium* was not affected after the 6 h exposure to the air emitted by the photocatalytic device in the case of the control test, where the photocatalytic device contained a honeycomb-shaped filter of only the gypsum without the photocatalyst composite and the same LED and fan as the other tests. Conversely, a significant growth reduction was observed already after 1 h of visible light illumination in the cases of both *B. subtilis* and *S. typhimurium*. After 2 h of treatment, almost no bacteria survived. By observing the change in the growth of *S. typhimurium* on agar plates at the beginning of the test versus at the end, the evident eradication of the bacteria is clearly exhibited in Figure 9a,b. The same is seen in the case of *B. subtilis*.

**Table 2:** Bacterial Viability^a^

Time [h]	*B. subtilis* [CFU] × 10^3^	*S. typhi* [CFU] × 103
0	1400	900
1	680	540
2	4	3
4	0.96	0.6
6	0.5	0.5

a The viability of *B. subtilis* and *S. typhimurium* as a function of time over remote photocatalysis induced by BiOCl_0.2_Br_0.8_.

The bactericidal activity of BiOCl_0.2_Br_0.8_ was also investigated under harsh conditions, that is, inside the refrigerator. *L. monocytogenes* is one of the most virulent foodborne pathogens that is stubborn, has the ability to grow at temperatures of up to 0°, and is not easily eliminated. Its *viability was tested* under cold conditions in the presence of our proposed system.

Two identical refrigerators containing two shelves each were utilized for performing the tests, one for the tests in the presence of the photocatalytic filter and another for the control tests where the photocatalytic device used contained a honeycomb-shaped filter of only the gypsum without the photocatalyst coating, with identical LED and fan as the other refrigerator’s device.

Microscope glass slides laid on empty Petri dishes and placed in two different locations inside the refrigerator (location “A” is the first refrigerator shelf and “B” is the shelf beneath) were contaminated using the bacterial suspension in both refrigerators (Figure S8).

BiOCl_0.2_Br_0.8_ started its activity after turning on both the LED light and the fan of the photocatalytic reactor.

At predetermined intervals, after the beginning of each test, the glass slides were taken out of the refrigerators, and the bacterial counting was performed after bacterial dilutions and growth via incubation using agar plates.

The test results are shown in Table 3. BiOCl_0.2_Br_0.8_ demonstrated an efficacious photocatalytic activity since the viability of *L. monocytogenes* decreased as a function of time after turning the system on, namely, activating the catalyst using the LED light and enriching the environment with oxidative radicals reaching the bacterium-contaminated surfaces. In both cases of the control and the actual tests, the concentration of *L. monocytogenes* at the beginning was 10^8^ CFU/g which did not change after 24 h of treatment; in the case of the control, however, the concentration reduced to less than 100 CFU/g after 12 h of treatment in the presence of the catalyst and to less than 10 CFU/g after 24 h.

**Table 3:** Viability of *L. monocytogenes* as a Function of Time

Location “A” (h)	*L. monocytogenes* [CFU/g]
0	1.2 × 10^8^
12	<100
24	<10
Location “B” (h)	*L. monocytogenes* [CFU/g]
0	1.2 × 10^8^
12	<100
24	<10
Control (without treatment) (h)	*L. monocytogenes* [CFU/g]
0	1.2 × 10^8^
24	1.0 × 10^8^

For full elimination of *L. monocytogenes*, one cycle was about 24 h of continuous photocatalytic deactivation where we could see almost full CFU count reduction from 10^8^ to less than 10 living bacteria.

### 3.3 Mechanistic Study

**Figure 10:**
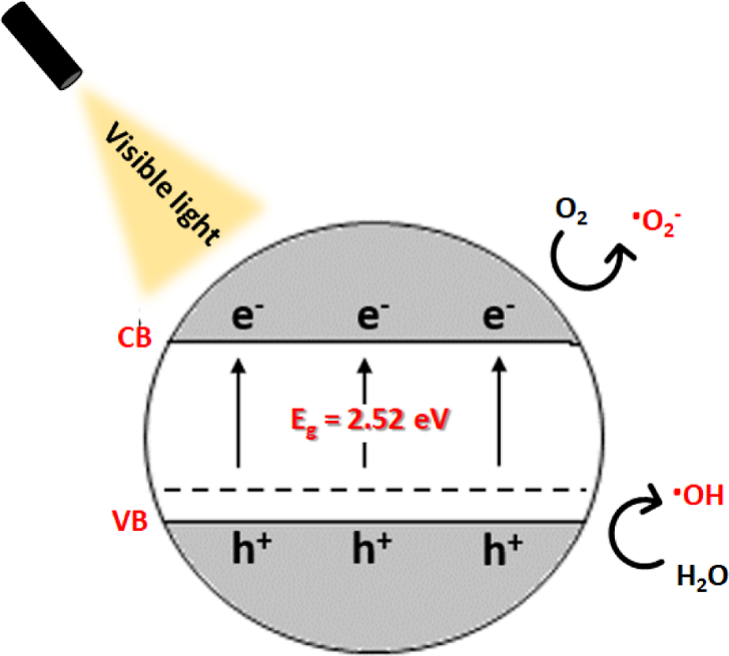
Schematic diagram for the photocatalytic mechanism of BiOCl_0.2_Br_0.8_ under visible light irradiation.

By comparing the VB edge potential of BiOCl_0.2_Br_0.8_ (Figure 10) to the standard redox potential of OH^•^/OH^–^ (1.99 eV), it is clear that the former has a more positive value, implying that the photogenerated hole is a stronger oxidant than the OH^•^ radical. The deep and positive VB (2.97 eV) indicates that water molecules in air can directly interact with these photogenerated holes, thus promoting the catalytic and accelerated production of hydroxyl radicals.

The purified air emitted from the photocatalytic reactor is enriched with reactive radicals (out-diffusion of oxidizing species) as elucidated here
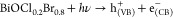
1
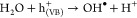
2
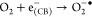
3

Specifically stating, being a radical, OH^•^ is generally recognized as a highly active species, and it is widely anticipated that the hydroxyl radical would also rapidly diffuse while interacting with water molecules (humidity in the air) via a hydrogen exchange reaction (analogous to the proton-exchange reaction). This phenomenon is described by the following chemical equation
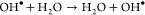
4

It is important to note that the free radical’s generation was also substantiated using hydrogen peroxide formation test strips. A test was performed (Figure S9) where the photocatalyst powder was activated by irradiation using a 300 W Xe lamp source after mixing with double-distilled water. After a couple of minutes of radiation, hydrogen peroxide was formed in the catalyst–water suspension with a concentration of 0.05–0.3 ppm. Hydrogen peroxide formation can only be explained by the presence of free radicals generated by the photocatalyst according to the reactions below
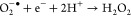

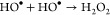


Both super oxide anion radicals and the hydroxyl radicals can react to form hydrogen peroxide; however, we believe that the second reaction is more likely to occur.

The photogenerated radicals (mainly hydroxyl radicals, as shown in our previous reports^17,27^) can diffuse through the air while interacting with water molecules and are continuously multiplied. In this way, when these radicals reach various contaminated surfaces, they fully sterilize them from any microorganism such as bacteria and viruses.

The microbial tests performed yielded satisfying results, both at room temperature and inside a refrigerator (4 °C). BiOCl_0.2_Br_0.8_ showed outstanding bactericidal activity which was expressed by a notable bacterial reduction on surfaces via remote visible light-driven photocatalysis (Figure S7). The ability to reduce bacterial levels across surfaces indicates a high capacity for reducing airborne contamination because no direct contact occurs between the photocatalyst and the bacteria that are located on surfaces, thus making the process more difficult and different from the case of aerosolized bacteria.

The selected bacteria pose a challenge because of their relatively high resistance capsule that increases the resistance of *S. typhimurium* and *B. subtilis*’s ability to form spores and the capability of *L. monocytogenes* to grow at temperatures of up to 0°.

BiOCl_0.2_Br_0.8_’s ability to eliminate bacteria, including ones that are biorecalcitrant, indicates its capacity to abolish viruses as well, for bacterial cells are way more complex than viruses, which are simple organisms made of a genetic material (DNA or RNA) and a protein coat (capsid).

Generally speaking, bromide-rich bismuth oxyhalides exhibit a higher antibacterial photocatalytic activity compared to that of bismuth oxychlorides. On the other hand, the care for selection of our proposed BiOCl_0.2_Br_0.8_ is also affected by the fact that a higher molar ratio of bromide shows stronger absorption of wavelengths in the visible light region due to narrower band gap values.

Thus, we strongly believe that BiOCl_0.2_Br_0.8_ is an excellent candidate for eliminating a wide range of contaminants including the coronavirus and many other harmful pathogens.

To the best of our knowledge, this is the first report demonstrating remote antibacterial activity using visible light without a direct contact between the catalyst coating and the bacterial species. Most, if not all, of the scientific research and studies investigate the direct molecular interactions between the tested bacteria and the developed photocatalyst. Herein, we shed light on an innovative concept which might create new opportunities in the field of applied materials specifically for indoor environmental cleanup.

## 4 Conclusions

In summary, the proposed bismuth oxyhalide semiconductor coated on a simple commercially available gypsum exhibited exceptional photocatalytic activity in the remote elimination of a plethora of highly resistant bacteria in diverse environments. This hybrid material is of a great potential in numerous applications including the construction industry, where it can be applied to interior walls of buildings, assuring everlasting sterile environments.

Another propitious application comprises the medical field, where the extended antibacterial effect of the studied composite can aid in biomaterials or implant coatings.

## Supporting Information Available

BiOCl_0.2_Br_0.8_ photocatalytic powder; honeycomb-shaped filter of the BiOCl_0.2_Br_0.8_@gypsum composites and the hexagonal-shaped mold; photocatalytic reactor; EDS elemental mapping images of the BiOCl_0.2_Br_0.8_ microsphere; SEM images acquired from the pure gypsum surface; nitrogen sorption isotherm of the BiOCl_0.2_Br_0.8_ photocatalytic powder; remote photocatalytic activity; different locations of the microscope glass slides used to perform the bacterial viability test under cold conditions; free radicals’ generation proof test via utilizing hydrogen peroxide detection strips; and EDS-coated surface atomic percentages (PDF)

## References

[ref1] Mitoraj D.; Jańczyk A.; Strus M.; Kisch H.; Stochel G.; Heczko P. B.; Macyk W. Visible Light Inactivation of Bacteria and Fungi by Modified Titanium Dioxide. Photochem. Photobiol. Sci. 2007, 6, 642–648. 10.1039/b617043a. 17549266

[ref2] Gamage Z.; Zhang J. Applications of Photocatalytic Disinfection. Int. J. Photoenergy 2010, 2010, 764870 10.1155/2010/764870.

[ref3] McCullagh K. J. A.; Edwards B.; Poon E.; Lovering R. M.; Paulin D.; Davies K. E. Intermediate Filament-like Protein Syncoilin in Normal and Myopathic Striated Muscle. Neuromuscular Disord. 2007, 17, 970–979. 10.1016/j.nmd.2007.06.004. 17629480

[ref4] Tongon W.; Chawengkijwanich C.; Chiarakorn S. Multifunctional Ag/TiO_2_/MCM-41 Nanocomposite Film Applied for Indoor Air Treatment. Build. Environ. 2014, 82, 481–489. 10.1016/j.buildenv.2014.09.014.

[ref5] Muranyi P.; Schraml C.; Wunderlich J. Antimicrobial Efficiency of Titanium Dioxide-Coated Surfaces. J. Appl. Microbiol. 2009, 108, 1966–1973. 10.1111/j.1365-2672.2009.04594.x. 19886892

[ref6] Gnayem H.; Dandapat A.; Sasson Y. Development of Hybrid BiOCl_*x*_Br_1-*x*_-Embedded Alumina Films and Their Application as Highly Efficient Visible-Light-Driven Photocatalytic Reactors. Chem.—Eur. J. 2016, 22, 370–375. 10.1002/chem.201503900. 26612508

[ref7] Kumar R.; Anandan S.; Hembram K.; Narasinga Rao T. Efficient ZnO-Based Visible-Light-Driven Photocatalyst for Antibacterial Applications. ACS Appl. Mater. Interfaces 2014, 6, 13138–13148. 10.1021/am502915v. 25029041

[ref8] Kim Y. J.; Gao B.; Han S. Y.; Jung M. H.; Chakraborty A. K.; Ko T.; Lee C.; Lee W. I. Heterojunction of FeTiO_3_ Nanodisc and TiO_2_ Nanoparticle for a Novel Visible Light Photocatalyst. J. Phys. Chem. C 2009, 113, 19179–19184. 10.1021/jp908874k.

[ref9] Bayan E. M.; Lupeiko T. G.; Pustovaya L. E.; Fedorenko A. G. Effect of Synthesis Conditions on the Photocatalytic Activity of Titanium Dioxide Nanomaterials. Nanotechnol. Russ. 2017, 12, 269–275. 10.1134/S199507801703003X.

[ref10] Chen H.; Gu Z.; An H.; Chen C.; Chen J.; Cui R.; Chen S.; Chen W.; Chen X.; Chen X.; Chen Z.; Ding B.; Dong Q.; Fan Q.; Fu T.; Hou D.; Jiang Q.; Ke H.; Jiang X.; Liu G.; Li S.; Li T.; Liu Z.; Nie G.; Ovais M.; Pang D.; Qiu N.; Shen Y.; Tian H.; Wang C.; Wang H.; Wang Z.; Xu H.; Xu J. F.; Yang X.; Zhu S.; Zheng X.; Zhang X.; Zhao Y.; Tan W.; Zhang X.; Zhao Y. Precise Nanomedicine for Intelligent Therapy of Cancer. Sci. China: Chem. 2018, 61, 1503–1552. 10.1007/s11426-018-9397-5.

[ref11] Park S. J.; Kang S. G.; Fryd M.; Saven J. G.; Park S. J. Highly Tunable Photoluminescent Properties of Amphiphilic Conjugated Block Copolymers. J. Am. Chem. Soc. 2010, 132, 9931–9933. 10.1021/ja1004569. 20608674

[ref12] Weng S.; Fang Z.; Wang Z.; Zheng Z.; Feng W.; Liu P. Construction of Teethlike Homojunction BiOCl (001) Nanosheets by Selective Etching and Its High Photocatalytic Activity. ACS Appl. Mater. Interfaces 2014, 6, 18423–18428. 10.1021/am5052526. 25330341

[ref13] Hu J.; Fan W.; Ye W.; Huang C.; Qiu X. Insights into the Photosensitivity Activity of BiOCl under Visible Light Irradiation. Appl. Catal., B 2014, 158–159, 182–189. 10.1016/j.apcatb.2014.04.019.

[ref14] Hu J.; Weng S.; Zheng Z.; Pei Z.; Huang M.; Liu P. Solvents Mediated-Synthesis of BiOI Photocatalysts with Tunable Morphologies and Their Visible-Light Driven Photocatalytic Performances in Removing of Arsenic from Water. J. Hazard. Mater. 2014, 264, 293–302. 10.1016/j.jhazmat.2013.11.027. 24316247

[ref15] He R.; Cao S.; Zhou P.; Yu J. Recent Advances in Visible Light Bi-Based Photocatalysts. Chin. J. Catal. 2014, 35, 989–1007. 10.1016/s1872-2067(14)60075-9.

[ref16] Ye L.; Deng K.; Xu F.; Tian L.; Peng T.; Zan L. Increasing Visible-Light Absorption for Photocatalysis with Black BiOCl. Phys. Chem. Chem. Phys. 2012, 14, 82–85. 10.1039/c1cp22876e. 22080233

[ref17] Gnayem H.; Sasson Y. Nanostructured 3D Sunflower-like Bismuth Doped BiOClxBr1-x Solid Solutions with Enhanced Visible Light Photocatalytic Activity as a Remarkably Efficient Technology for Water Purification. J. Phys. Chem. C 2015, 119, 19201–19209. 10.1021/acs.jpcc.5b05217.

[ref18] Maile F. J.; Pfaff G.; Reynders P. Effect Pigments - Past, Present and Future. Prog. Org. Coat. 2005, 54, 150–163. 10.1016/j.porgcoat.2005.07.003.

[ref19] Kijima N.; Matano K.; Saito M.; Oikawa T.; Konishi T.; Yasuda H.; Sato T.; Yoshimura Y. Oxidative Catalytic Cracking of N-Butane to Lower Alkenes over Layered BiOCl Catalyst. Appl. Catal., A 2001, 206, 237–244. 10.1016/S0926-860X(00)00598-6.

[ref20] Ye L.; Su Y.; Jin X.; Xie H.; Zhang C. Recent Advances in BiOX (X = Cl, Br and I) Photocatalysts: Synthesis, Modification, Facet Effects and Mechanisms. Environ. Sci.: Nano 2014, 1, 90–112. 10.1039/c3en00098b.

[ref21] Chang X.; Huang J.; Cheng C.; Sui Q.; Sha W.; Ji G.; Deng S.; Yu G. BiOX (X = Cl, Br, I) Photocatalysts Prepared Using NaBiO_3_ as the Bi Source: Characterization and Catalytic Performance. Catal. Commun. 2010, 11, 460–464. 10.1016/j.catcom.2009.11.023.

[ref22] Zhang X.; Ai Z.; Jia F.; Zhang L. Generalized One-Pot Synthesis, Characterization, and Photocatalytic Activity of Hierarchical BiOX (X = Cl, Br, I) Nanoplate Microspheres. J. Phys. Chem. C 2008, 112, 747–753. 10.1021/jp077471t.

[ref23] Li T. B.; Chen G.; Zhou C.; Shen Z. Y.; Jin R. C.; Sun J. X. New Photocatalyst BiOCl/BiOI Composites with Highly Enhanced Visible Light Photocatalytic Performances. Dalton Trans. 2011, 40, 6751–6758. 10.1039/c1dt10471c. 21617792

[ref24] Wang W.; Huang F.; Lin X. xBiOI-(1 - x)BiOCl as Efficient Visible-Light-Driven Photocatalysts. Scr. Mater. 2007, 56, 669–672. 10.1016/j.scriptamat.2006.12.023.

[ref25] Wang W.; Huang F.; Lin X.; Yang J. Visible-Light-Responsive Photocatalysts xBiOBr-(1-x)BiOI. Catal. Commun. 2008, 9, 8–12. 10.1016/j.catcom.2007.05.014.

[ref26] Shenawi-Khalil S.; Uvarov V.; Kritsman Y.; Menes E.; Popov I.; Sasson Y. A New Family of BiO(Cl_*x*_Br_1-*x*_) Visible Light Sensitive Photocatalysts. Catal. Commun. 2011, 12, 1136–1141. 10.1016/j.catcom.2011.03.014.

[ref27] Gnayem H.; Sasson Y. Hierarchical Nanostructured 3D Flowerlike BiOCl_*x*_Br_1-*x*_ Semiconductors with Exceptional Visible Light Photocatalytic Activity. ACS Catal. 2013, 3, 186–191. 10.1021/cs3005133.

[ref28] Liu Y.; Son W. J.; Lu J.; Huang B.; Dai Y.; Whangbo M. H. Composition Dependence of the Photocatalytic Activities of BiOCl_1-*X*_Br_*x*_ Solid Solutions under Visible Light. Chem.—Eur. J. 2011, 17, 9342–9349. 10.1002/chem.201100952. 21732448

[ref29] Bhachu D. S.; Moniz S. J. A.; Sathasivam S.; Scanlon D. O.; Walsh A.; Bawaked S. M.; Mokhtar M.; Obaid A. Y.; Parkin I. P.; Tang J.; Carmalt C. J. Bismuth Oxyhalides: Synthesis, Structure and Photoelectrochemical Activity. Chem. Sci. 2016, 7, 4832–4841. 10.1039/c6sc00389c. 30155131 PMC6016733

[ref30] Shen F.; Zhou L.; Shi J.; Xing M.; Zhang J. Preparation and Characterization of SiO_2_/BiOX (X = Cl, Br, I) Films with High Visible-Light Activity. RSC Adv. 2015, 5, 4918–4925. 10.1039/c4ra10227d.

[ref31] Imam S. S.; Adnan R.; Mohd Kaus N. H.; Hussin M. H. Room-Temperature Synthesis of Bi/BiOBr Composites for the Catalytic Degradation of Ciprofloxacin Using Indoor Fluorescent Light Illumination. J. Mater. Sci.: Mater. Electron. 2019, 30, 6263–6276. 10.1007/s10854-019-00930-z.

[ref32] Hussain M. B.; Khan M. S.; Loussala H. M.; Bashir M. S. The Synthesis of a BiOCl_*x*_Br_1-*x*_ Nanostructure Photocatalyst with High Surface Area for the Enhanced Visible-Light Photocatalytic Reduction of Cr(VI). RSC Adv. 2020, 10, 4763–4771. 10.1039/c9ra10256f. 35495237 PMC9049196

[ref33] Song J. M.; Mao C. J.; Niu H. L.; Shen Y. H.; Zhang S. Y. Hierarchical Structured Bismuth Oxychlorides: Self-Assembly from Nanoplates to Nanoflowers via a Solvothermal Route and Their Photocatalytic Properties. CrystEngComm 2010, 12, 3875–3881. 10.1039/c003497p.

