# Supplementary material for: Remote Photocatalytic Eradication of Biorecalcitrant Microorganisms via BiOCl0.2Br0.8—The Applied Aspects of Visible Light-Driven Photocatalysis
Source: ACS Omega. 2022 Aug 15;7(34):29625–33. doi: 10.1021/acsomega.2c01502 (PMC9434757; doi:10.1021/acsomega.2c01502)
Supplement: Supplementary file 1 — ao2c01502_si_001.pdf [file ao2c01502_si_001.pdf]

Remote Photocatalytic Eradication of Bio-recalcitrant Microorganisms via $BiOCl_{0.2}Br_{0.8}$ - The Applied Aspects of Visible Light Driven Photocatalysis

Razan Abbasi, Hani Gnayem and Yoel Sasson*.*

Razan Abbasi - Casali Center of Applied Chemistry, Institute of Chemistry, The Hebrew University of Jerusalem, 9190401, Israel.

Email: Razan.abbasi@mail.huji.ac.il

Prof. Yoel Sasson - Casali Center of Applied Chemistry, Institute of Chemistry, The Hebrew University of Jerusalem, 9190401, Israel.

Email: ysasson@huji.ac.il

Dr. Hani Gnayem – Casali Center of Applied Chemistry, Institute of Chemistry, The Hebrew University of Jerusalem, 9190401, Israel.

Email: Hani.gnayem@mail.huji.ac.il

Supporting Information

Photocatalytic powder. A digital photo of $BiOCl_{0.2}$ $Br_{0.8}$ powder is shown in Figure S1.

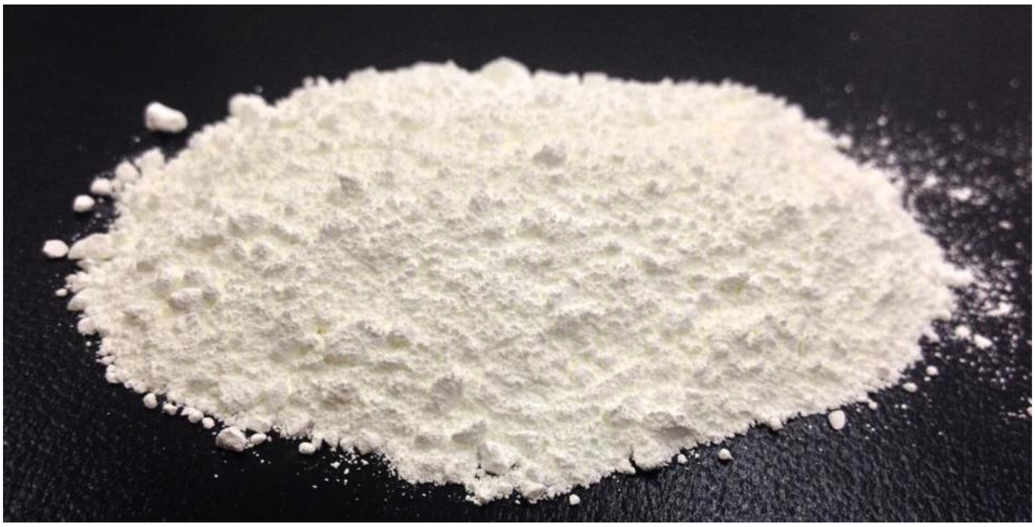

Figure S1. $BiOCl_{0.2}$ $Br_{0.8}$ Photocatalytic powder.

Gypsum composites. The honeycomb shaped filter of $BiOCl_{0.2}Br_{0.8}$ @ gypsum composites is shown in figure S2.

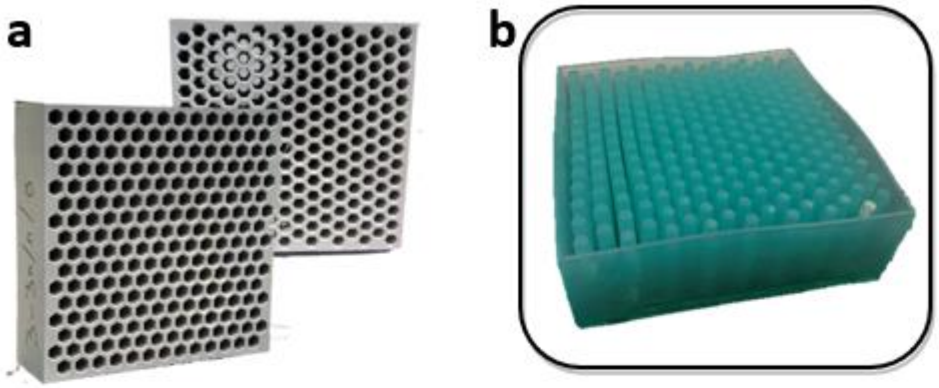

Figure S2. Honeycomb shaped filter of the $BiOCl_{0.2}Br_{0.8}$ @ gypsum composites (a), and the hexagonal shaped mold (b).

Photocatalytic reactor. The photocatalytic reactor used is shown in Figure S3.

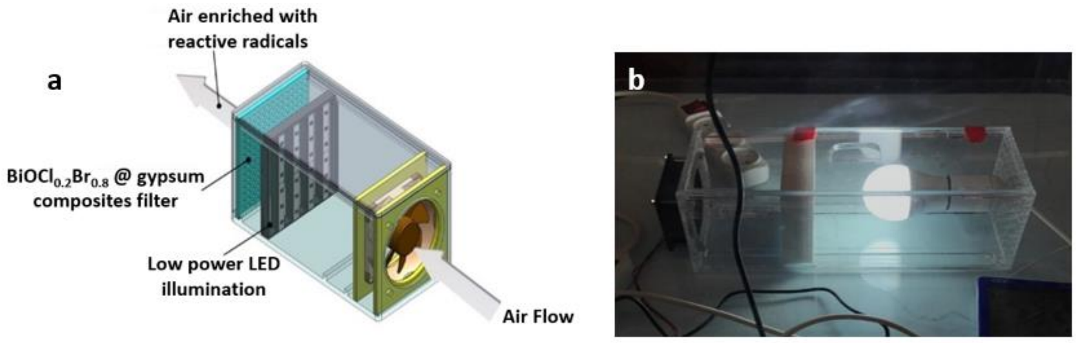

Figure S3. Photocatalytic reactor. Schematic figure (a), and the devised and used reactor (b).

EDS. EDS atomic percentages of coated surface at 30 kV accelerating voltage are shown in Table S1.

Element	Atomic %
C	10.81
O	45.4
Si	0.31
S	7.5
Ca	5.9
Cl	1.48
Bi	23.42
Br	5.18
Total	100

Table S1. EDS coated surface atomic percentages.

EDS elemental mapping. EDS elemental mapping images of $BiOCl_{0.2}Br_{0.8}$ microsphere are shown in Figure S4.

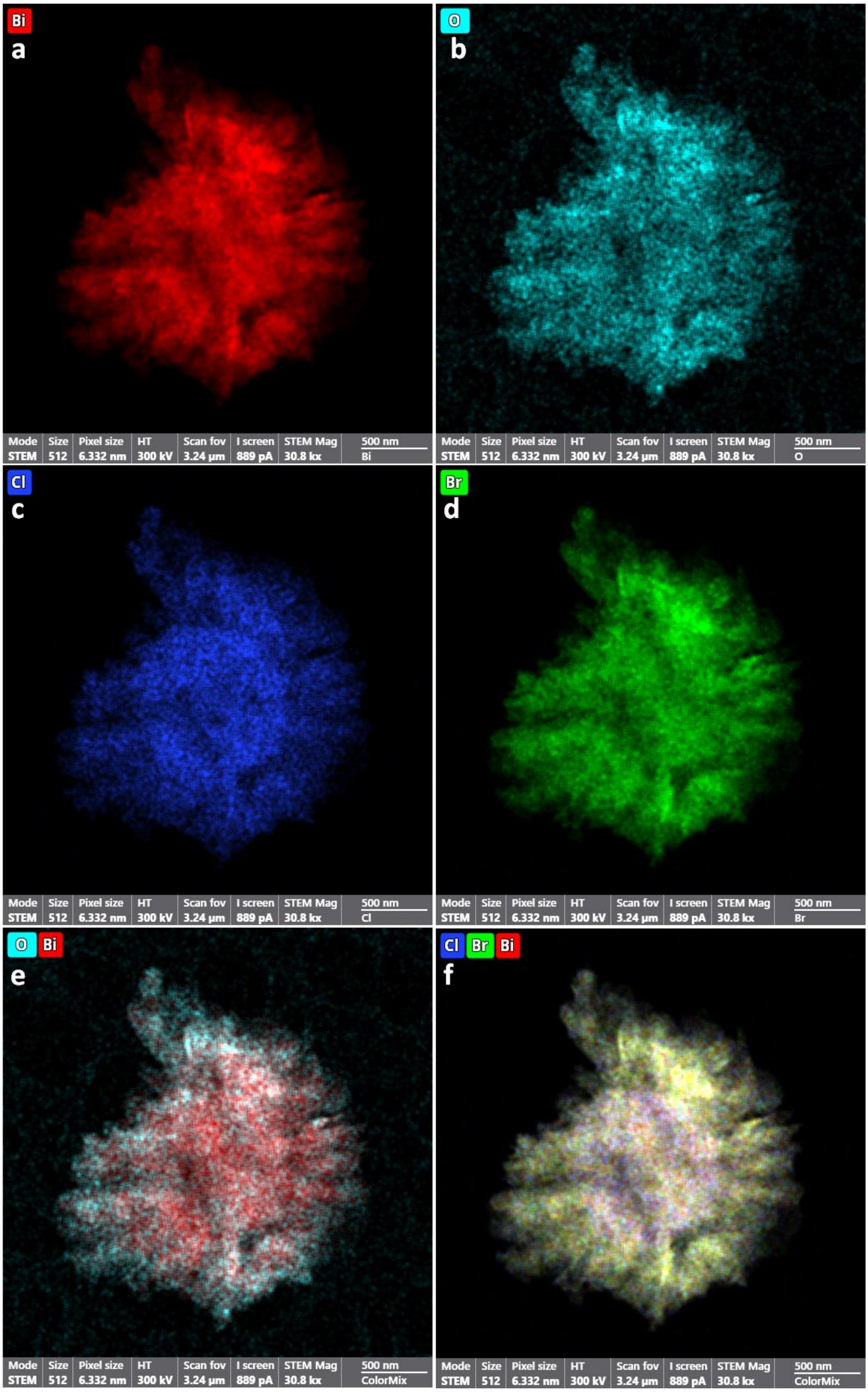

Figure S4. EDS elemental mapping images of **$BiOCl_{0.2}$ $Br_{0.8}$** microsphere. The red, turquoise, blue and green areas correspond to the elements of Bi, O, Cl, and Br, respectively (a, b, c and d). Overlay of: Bi and O is shown in (e), Bi, Cl and Br in (f).

SEM images. SEM images acquired from pure gypsum are shown in Figure S5.

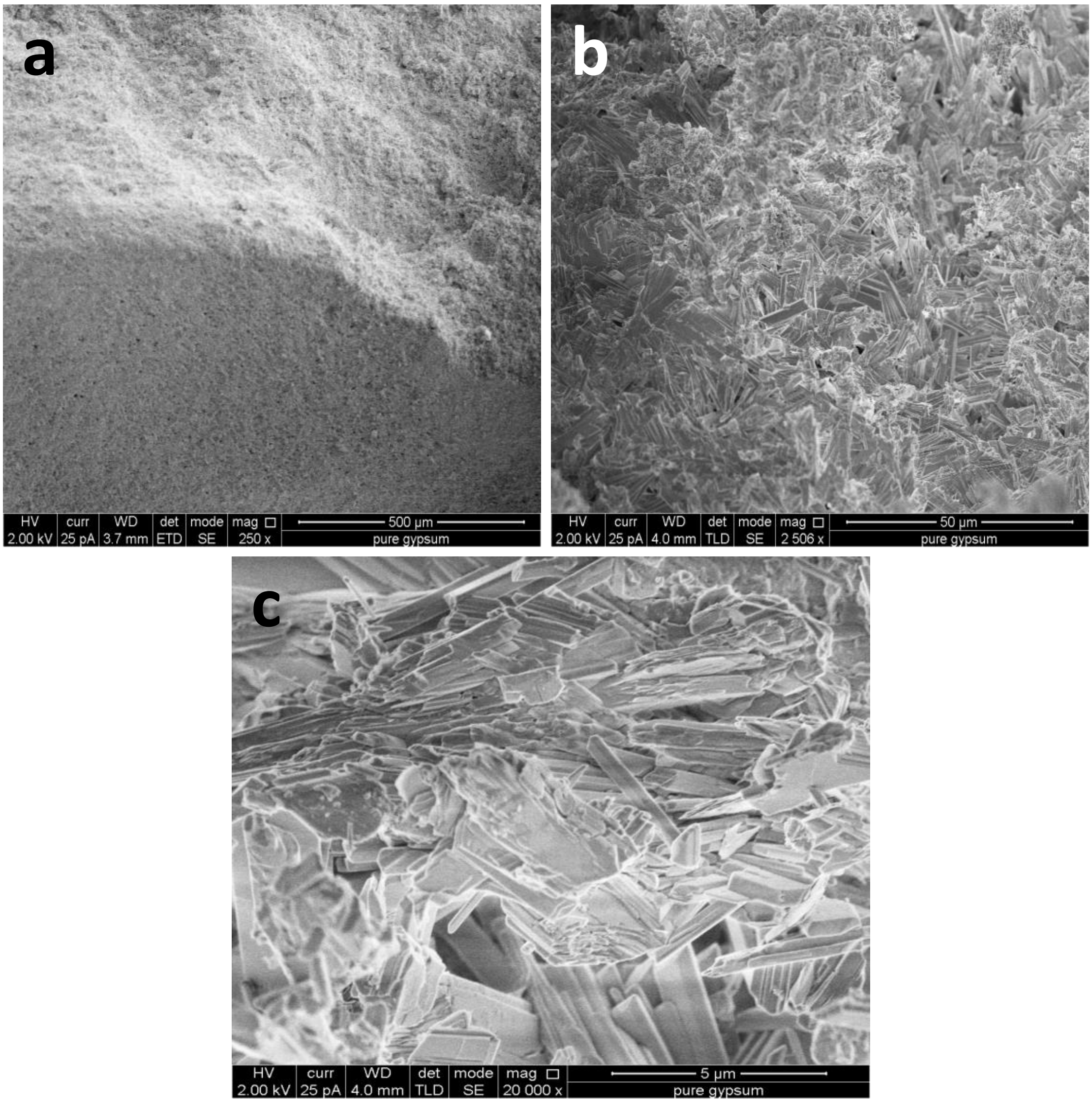

Figure S5. SEM images acquired from pure gypsum surface (a, b, and c).

BET surface area. Nitrogen sorption isotherm of pure $BiOCl_{0.2}Br_{0.8}$ powder is shown in Figure S6.

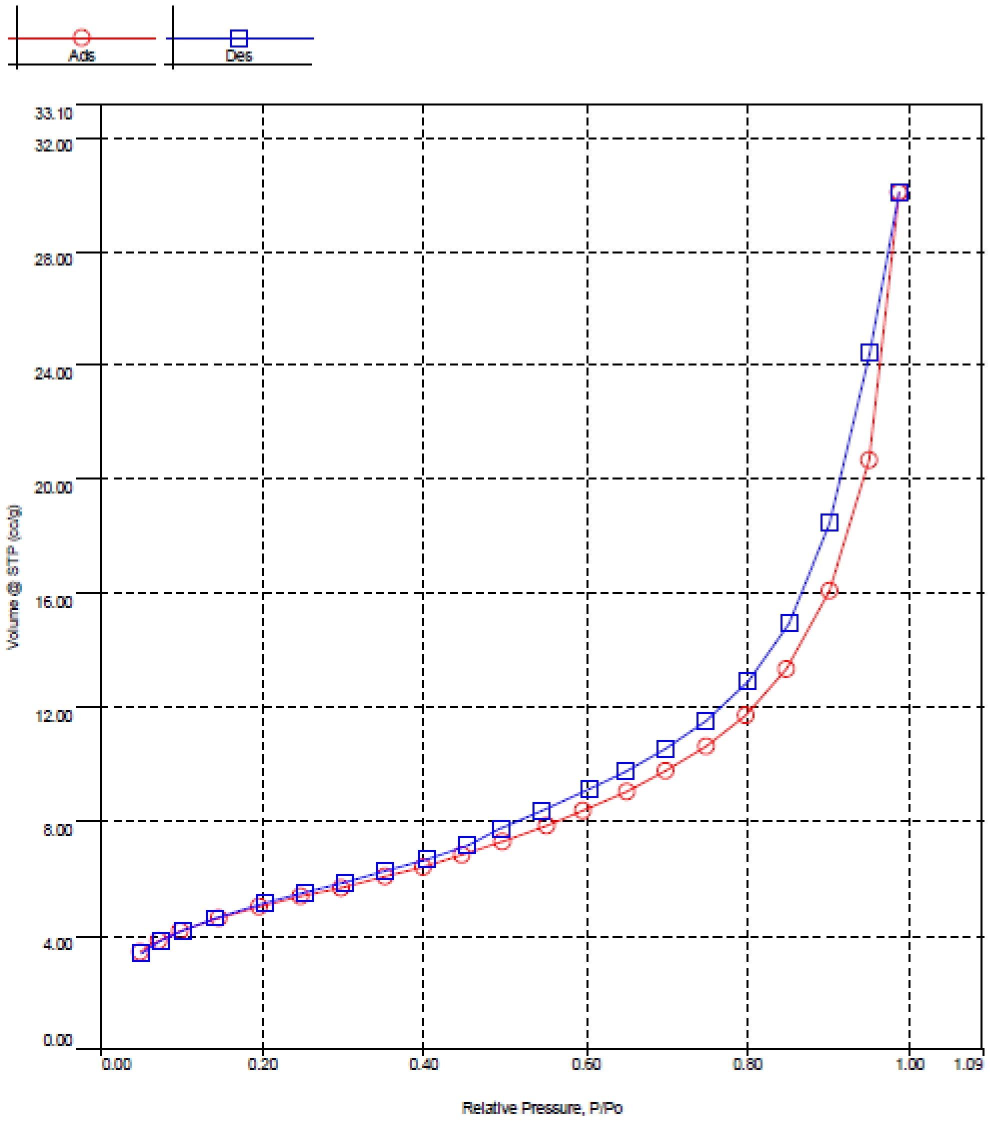

Figure S6. Nitrogen sorption isotherm of $\mathbf{BiOCl_{0.2}\ Br_{0.8}}$ Photocatalytic powder

***Remote photocatalysis*.** The remote photocatalytic process is shown in Figure S7.

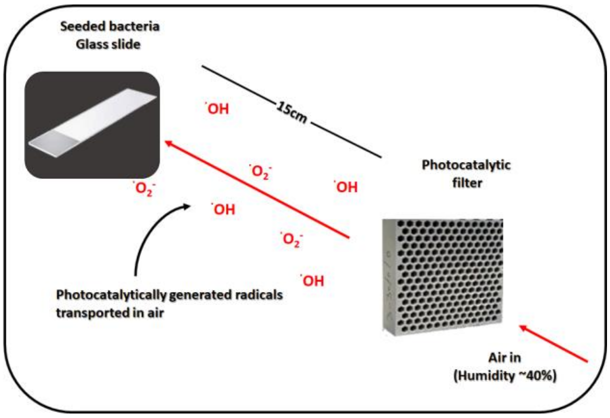

Figure S7. Remote photocatalytic activity.

***Listeria monocytogenes* test location.** The refrigerator used to test the bacterial viability in cold conditions is shown in Figure S8.

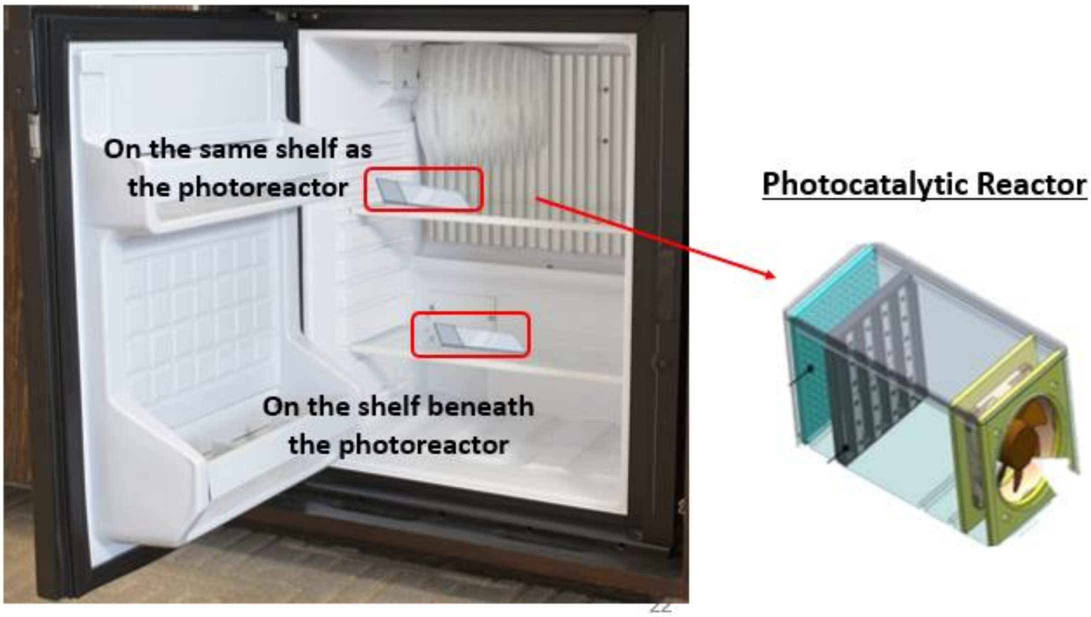

Figure S8. The different locations of the microscope glass slides used to perform the bacterial viability test in cold conditions.

Free radicals' generation proof. Hydrogen peroxide formation test using hydrogen peroxide detection strips is shown in Figure S9.

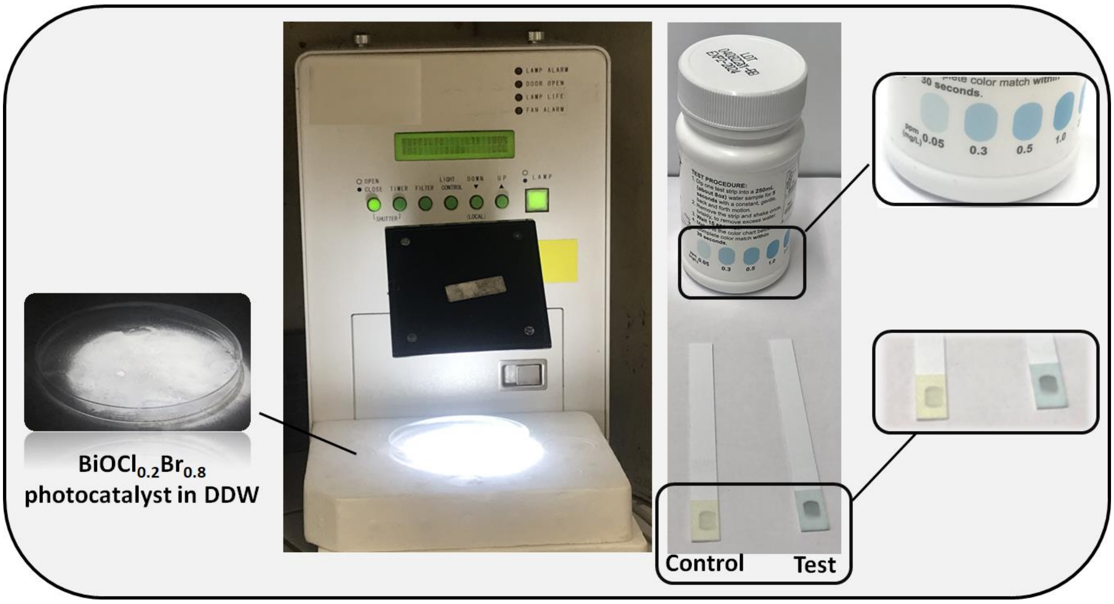

Figure S9. Free radicals' generation proof test via utilizing hydrogen peroxide detection strips.
